# Impacts of COVID-19 on alcohol use among help-seeking adults

**DOI:** 10.3389/adar.2023.11159

**Published:** 2023-05-15

**Authors:** Aradhana Srinagesh, Sarah Forthal, Sean P. Madden, L. A. R. Stein, Frederick Muench

**Affiliations:** ^1^ Department of Psychology, University of Rhode Island, South Kingston, RI, United States; ^2^ Partnership to End Addiction, New York, NY, United States; ^3^ Feinstein Institutes for Medical Research, Northwell Health, Manhasset, NY, United States

**Keywords:** COVID-19, pandemic, adult, alcohol, moderation

## Abstract

The coronavirus (COVID-19) pandemic has been associated with both increased and decreased alcohol use. Authors explored reasons for increased and decreased alcohol use since the COVID-19 lockdown (March 2020) in a sample of help-seeking adults (HSA) participating in a remote-based alcohol reduction text-messaging intervention in the USA. At the time of recruitment, the HSA in this study were interested in reducing rather than stopping their alcohol consumption. An optional self-report questionnaire was completed by 324 participants (mean age 41.6 ± 10.2 years; 71.5% female; 83.9% White) in February 2021. Survey questions assessed sociodemographic factors, social stressors (quarantine conditions, employment status, changes to daily routine), and drinking patterns. Authors fit two ordinal logistic regression models: one for increased drinking and one for decreased drinking, as functions of the potential predictors and control variables. Most participants (*n* = 281; 87.0%) reported drinking more than usual since COVID-19 lockdown began. The most common self-reported reasons for drinking more were increased stress/anxiety (74.7%), boredom (69.4%), and spending more time at home (65.5%) whereas reasons for drinking less were less socializing (33.7%) and worrying about how alcohol would impact the immune system (31.5%). Identifying as female, severity of changes to daily routine, and increased access to alcohol were significantly associated with drinking more than usual. These data suggest that the general consequences of the pandemic in the general population (e.g., boredom) led to greater alcohol use among help-seeking adults attempting to reduce their drinking. Identifying these factors may help create more targeted interventions during public health crises.

## Introduction

Excessive alcohol consumption is associated with various health risks and societal challenges [1, 2]. The health and economic hardships of the COVID-19 pandemic are comparable to or perhaps greater than those of past natural or environmental disasters [3, 4]. Previous literature suggests when individuals experience periods of economic or psychological stress, they may consume more alcohol, resulting in increased symptoms of alcohol abuse and dependence [5–9]. In the winter and spring of 2020, governments across the world declared a state of emergency and took drastic measures (e.g., physical distancing, closing businesses, banning large gatherings, quarantining, etc.) to help mitigate the spread of COVID-19. Past literature would suggest that the contextual changes resulting from the measures taken would suggest an increase in harmful alcohol use. However, the current literature on COVID-19-related drinking patterns remains varied, indicating both increased and decreased alcohol consumption [10–13], or no changes at all [1, 14, 15].

Emerging evidence from national and global surveys conducted during the peak of the pandemic (March 2020) shows increased rates of substance use, specifically alcohol use [16]. Preliminary reports suggest, amid COVID-19, both alcohol sales and consumption have increased [17, 18], potentially in response to the use of alcohol as a coping mechanism for psychological distress [18]. For instance, a recently published study on alcohol consumption during the pandemic in the United States (US) showed that Americans drank about 14% more alcohol in 2020, and in a report of national sales of alcohol, online sales increased 262% in 2020, compared to 2019 [19]. Despite alcohol sales not remaining at these levels, overall data for the week ending on 21 March 2020 indicated that online alcohol sales increased by 234% in the US [19].

Several factors have been posited about the increase in alcohol volume sales. Specifically, individuals may have been stockpiling due to concern over inaccessibility during the pandemic’s early phases, which may have inflated these trends; it is likely that the increase in sales was at least partly related to an increase in consumption. Increased accessibility to alcohol is also a known risk factor for increased alcohol use [20, 21]. The negative impact of the COVID-19 pandemic on financial health and social functioning may have increased levels of psychological distress, social isolation, income insecurity, lack of work boundaries due to working from home, and job loss, contributing to increased alcohol consumption [12, 22–29]. Additional factors associated with increased alcohol consumption during the lockdown period included reduced healthcare access, increased access to alcohol delivery services, parental status (i.e., parent versus non-parent), less social connectedness, and increased levels of depression [28, 30, 31].

Conversely, reduced availability and affordability of alcoholic beverages, lack of social gatherings, whether alcohol was considered an essential item, financial distress, and closure of bars were associated with reduced alcohol consumption [12, 32–34]. These findings suggest that restrictions limiting access to alcohol, particularly outside the home (e.g., social gatherings, bars, happy hour, and pubs) might have led to lower alcohol use in certain populations. Additionally, one study suggests that alcohol consumption during COVID-19 decreased among men, while it remained steady among women [35]. This may have been due to broad gender differences in the relationship between loneliness and alcohol use: For instance, feelings of loneliness experienced early in the pandemic were associated with increased alcohol problems for women and a slow decline in alcohol use over time for men. The varying evidence suggests that the relationship between alcohol consumption and COVID-19 is more nuanced, possibly affecting distinct subpopulations in diverse ways. Some variables have been related to both increased and decreased use. For instance, a plausible rationale may be that some individuals were social drinkers, in which case they would decrease their use. On the other hand, some may prefer to drink alone and so their drinking increased.

In the present study, we sought to examine increases and decreases in alcohol use in help-seeking adults (HSA) with at-risk drinking, who may have viewed the pandemic as an opportunity to reduce their drinking and meet their drinking goals. Specifically, the HAS in this study were looking to reduce their drinking via text messaging intervention, rather than through traditional treatment (i.e., Alcoholics Anonymous, in-patient, outpatient).

The National Institute on Alcohol Abuse and Alcoholism (NIAAA) defines at-risk drinking as drinking above the recommended drinking guidelines (i.e., ≤3 standard drinks in one sitting or ≤7 per week for women, and ≤4 standard drinks in one sitting or ≤14 per week for men) without meeting criteria for more severe alcohol use disorders (AUD). At-risk drinkers are at a higher risk of experiencing negative consequences related to drinking along with the risk of progressing to an AUD [36, 37].

Although research finds that many individuals reported changing their drinking during the pandemic [33, 38, 39], there are currently no published data on the impacts of COVID-19 on alcohol consumption among individuals who were seeking to reduce their drinking prior to the pandemic. The present study explores potential predictors of changes in drinking among HSA participating in an alcohol reduction text-messaging intervention. As COVID-19 continues to evolve, understanding the immediate and long-term impacts of alcohol consumption among vulnerable populations can inform appropriate clinical and policy interventions.

## Materials and methods

### Procedure

Participants were recruited from our primary study (Muench et al., under revision), a randomized controlled trial of mobile messaging interventions for HSA with at-risk drinking, which recruited prospective participants primarily via social media outlets such as Facebook or online alcohol screening/help-seeking sources such as AlcoholScreening.org. This study was approved by both the Feinstein Institutes for Medical Research and Solutions IRB, and clinical investigations were conducted according to the principles expressed in the Declaration of Helsinki. All participants provided electronic informed consent. To meet the eligibility criteria, participants had to consume at least 13 and 15 standard drinks per week for women and men, respectively, which was later modified to include 9 and 11 standard drinks per week for women and men, respectively. Recruitment commenced in March 2019, prior to the pandemic; however, the vast majority of participants enrolled the following year in March 2020, during the pandemic (*n* = 671; 92%). For the present study, on 5 February 2021, study staff invited all 731 participants via text message to complete an optional cross-sectional questionnaire about their alcohol use since COVID-19 lockdown began. A total of 403 responses were received. Duplicate entries were discarded (*n* = 64; keeping the first or most complete response), as well as blank entries (*n* = 3), and data that could not be linked to a trial participant (*n* = 12), resulting in a final sample size of 324.

### Measures

The questionnaire (11 questions) was administered via REDCap [40], a secure web-based application, and assessed lockdown-related effects and behaviors (compliance with social distancing and changes to employment status, fear of contracting COVID-19, and mental health implications, daily routine, and alcohol supply). We assessed changes in drinking since lockdown began through 4 questions in March of 2020. Sociodemographic data (age, sex, race, marital status, and education level) were collected previously when participants enrolled in the primary study. The questionnaire was open for 7 days, and a reminder text was sent to complete it on 12 February 2021. Participants did not receive compensation for completing the questionnaire.

Questions assessing the impacts of COVID-19 were adapted from The Pandemic Stress Index [41], Coronavirus Impact Scale [42], and COVID-19 Community Response Survey [43]. The Resource Portal created by NIH was utilized to find COVID-19-specific survey tools [44].

Compliance with social distancing was assessed by asking: 1) “Have you practiced social distancing? (i.e., reduced your physical contact with people outside of your home in social, work, or school settings by avoiding large groups and staying 3–6 feet away from other people when out in public)” and 2) “Do you regularly wear a mask when outside of your house or pod*? (*a group of people from different households who agree to social distance from other people but not each other).”

Self-reported changes in drinking since the COVID-19 lockdown began were assessed with the question: “Have you experienced any of the following because of COVID-19 (since COVID-19 lockdown began)?” Respondents selected the frequency at which they “drank more than usual” and “drank less than usual” (very frequently, frequently, occasionally, rarely, never, or prefer not to say). Responses were coded as ordinal variables ranging from 1 (never) to 5 (very frequently). Respondents who indicated they drank more or less than usual also reported their perceived reasons for doing so. Possible reasons for drinking more were: increased stress/anxiety, boredom, spending more time at home, easily becoming a part of the routine, worrying about COVID-19 and/or the future, enjoying drinking more, working additional hours, being dependent on alcohol, and other sources. Possible reasons for drinking less were: decreased stress/anxiety, less socializing, worrying about how alcohol would impact the immune system, worrying about becoming ill from COVID-19, having diagnosed or suspected COVID-19, working additional hours, having financial concerns, enjoying drinking less, and other. Participants could select multiple reasons and were prompted to “select more than one option if it applies.” Question and response options were adapted from the survey that Grossman et al. [24] used in their study. Frequency of alcohol use was also assessed by asking: “Since COVID-19 lockdown began, how often did you usually have any kind of drink containing alcohol? By a drink we mean half an ounce of absolute alcohol (e.g., a 12-ounce can or glass of beer or cooler, a 5-ounce glass of wine, or a drink containing 1 shot of liquor). Choose only one.” with the following answer choices: every day, 5 to 6 times a week, 3 to 4 times a week, twice a week, once a week, 2 to 3 times a month, once a month, 3 to 11 times in the past year, and 1 or 2 times in the past year.

Potential predictors of changes in drinking included lockdown-related (compliance with social distancing and changes to employment status, daily routine, and alcohol supply) and sociodemographic (age, sex, race, marital status, and education level) variables, all self-reported and categorized as shown in Table 1. Authors also included as control variables the number of days each participant had been in the intervention trial, calculated as the difference between their baseline and COVID-19 questionnaire dates, and the type of intervention received as part of the trial.

**TABLE 1:** Questionnaire responses and their associations with changes in drinking (N = 324).

Characteristic	Mean (SD)	OR (95% CI), drank more^a^	OR (95% CI), drank less^a^
Age (N = 308)	41.6 (10.1)	1.00 (0.97, 1.02)	0.98 (0.95, 1.00)

	N (%)	OR (95% CI), drank more^a^	OR (95% CI), drank less^a^
Sex			
Male	92 (28.4)	REF	REF
Female	232 (71.6)	1.21 (0.74, 1.98)	0.87 (0.53, 1.43)
Race			
White	272 (84.0)	REF	REF
Non-white^b^	52 (16.1)	0.56 (0.31, 1.04)	1.28 (0.68, 2.41)
Marital status			
Single (never married)	86 (26.5)	REF	REF
Married	159 (49.1)	0.64 (0.37, 1.12)	0.88 (0.51, 1.52)
Separated, divorced, or widowed	79 (24.4)	0.89 (0.46, 1.73)	0.93 (0.49, 1.79)
Education level			
Associates degree or less	87 (26.9)	REF	REF
Bachelor’s degree or more	237 (73.2)	1.24 (0.74, 2.10)	0.61 (0.36, 1.03)
Have you practiced social distancing?			
No, I do not social distance	42 (13.2)	REF	REF
Yes, I have been completely quarantined (alone or with family)	44 (13.6)	**0.33 (0.14, 0.82)**	1.22 (0.51, 2.91)
Yes, I have seen others indoors but not been within about six feet of individuals outside my household	125 (38.6)	**0.38 (0.18, 0.79)**	1.44 (0.70, 2.94)
Yes, I have seen others outdoors but not been within about six feet of individuals outside my household	112 (34.6)	**0.29 (0.14, 0.63)**	1.46 (0.69, 3.07)
Has your employment status been affected by COVID-19?			
No, I am able to work from home	117 (36.1)	REF	REF
No, I was not working before	21 (6.5)	1.02 (0.38, 2.76)	0.62 (0.23, 1.67)
No, I am working same as before	139 (42.9)	0.76 (0.46, 1.25)	0.97 (0.59, 1.59)
Yes, I am currently unemployed due to COVID	47 (14.5)	0.53 (0.26, 1.10)	**0.41 (0.20, 0.84)**
Have your daily routines changed since COVID lockdown began?			
No, I have had no changes to my routines	11 (3.4)	REF	REF
Yes, I have had mild changes to a few of my routines	60 (18.5)	1.91 (0.60, 6.09)	1.64 (0.50, 5.43)
Yes, I have had moderate changes across several of my routines	123 (38.0)	3.67 (1.18, 11.37)	0.94 (0.30, 2.99)
Yes, I have had severe changes across most or all of my routines	130 (40.1)	7.29 (2.26, 23.50)	0.88 (0.27, 2.88)
Has your access to alcohol been affected by COVID-19?			
No	166 (51.2)	REF	REF
Yes, my supply has increased (i.e., due to buying in bulk)	126 (38.9)	**4.19 (2.54, 6.91)**	**0.41 (0.25, 0.67)**
Yes, my supply has decreased	32 (10.0)	**0.15 (0.07, 0.33)**	**11.00 (4.85, 25.00)**

^a^ Estimated using ordinal logistic regression models that included all variables in the table, plus the number of days each participant had been in an intervention trial and type of intervention received.

^b^ Includes participants who identified as Asian/Pacific Islander, Black/African American, Mixed, Native American, and Other race.

Bolded values are statistically significant at *p* < 0.05.

SD, standard deviation; OR, odds ratio; CI, confidence interval; REF, reference group.

### Statistical analysis

Questionnaire results were reported using frequencies and percentages for categorical variables and means and standard deviations for continuous variables. To explore independent predictors of changes in drinking since COVID-19 lockdown, authors fit two ordinal logistic regression models: one for increased drinking and one for decreased drinking, as functions of the potential predictors and control variables. Regression coefficients for each potential predictor were exponentiated and presented as odds ratios. Each odds ratio represents the proportional increase in odds of indicating a higher frequency of drinking more or less than usually associated with a one-unit change in the predictor, holding other model variables constant. Missing data was handled using listwise deletion. REDCap version 12.2.10,40) was used for data management and SAS version 9.4 [45] and Stata version 15 [46] for data cleaning and analyses.

## Results

### Sociodemographic variables

The sample was predominantly female-identifying (71.6%), White (84.0%), and had bachelor’s degrees or higher (some graduate school or a graduate degree; 73.2%). Participants’ average age was 41.6 years (SD = 10.2; range = 21–75). Roughly half (49.1%) of participants were currently married, and the remaining reported being single and never married (25.5%), or divorced, separated, or widowed (24.4%).

### COVID-19-related variables

Most participants practiced social distancing (86.8%), remained employed (79.0%, including 36.1% working from home), and experienced changes to their routines (96.6%). Approximately half of the participants (*n* = 158) reported changes in their supply of alcohol, with 38.9% having increased access and 10.0% having decreased access (Table 1).

**FIGURE 1:**
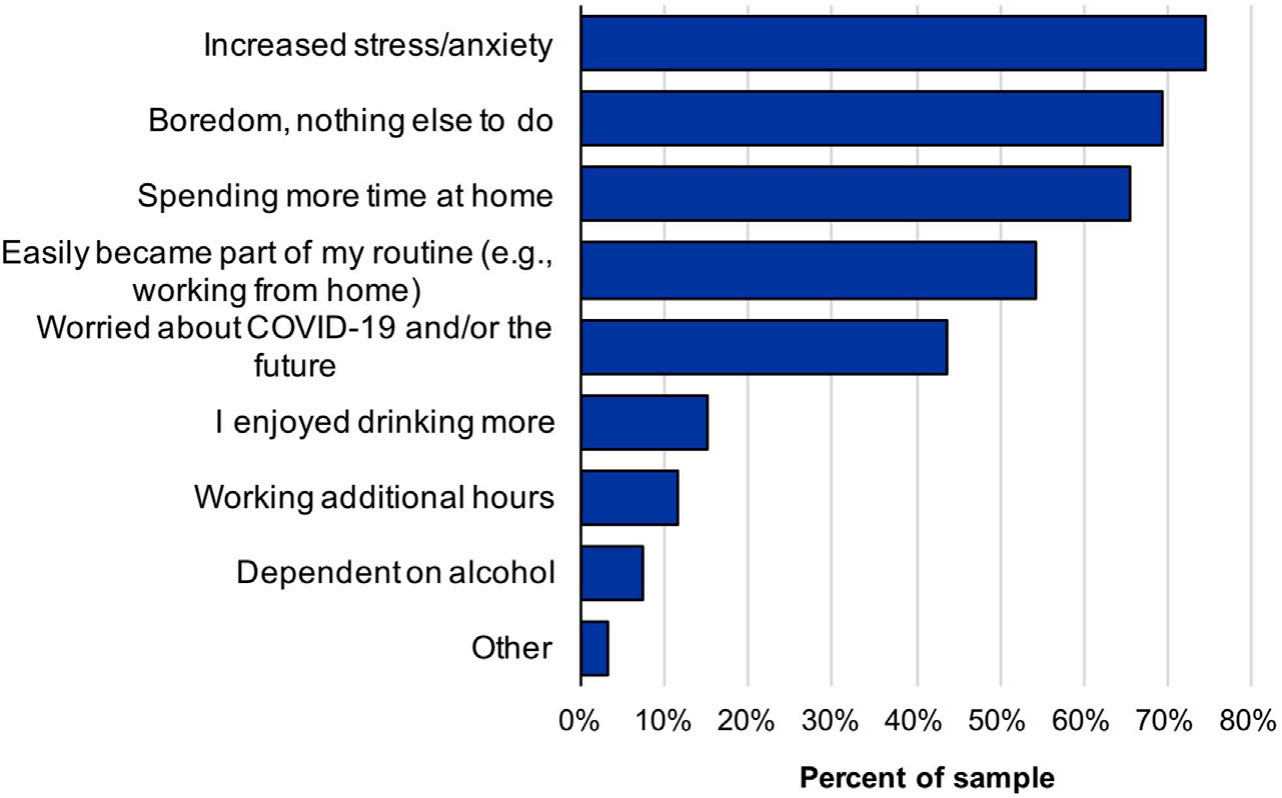
Reasons participants drank more than usual during the COVID-19 pandemic (N = 281).

Most participants (*n* = 281) reported drinking more than usual since the COVID-19 lockdown began (22.2% very frequently, 35.8% frequently, 26.9% occasionally, 10.5% rarely, and 4.6% never). The most common self-reported reasons for drinking more were increased stress/anxiety (74.7%), boredom (69.4%), and spending more time at home (65.5%) (Figure 1). Statistically significant predictors of drinking more than usual were not social distancing, experiencing more severe changes to daily routines, and increased access to alcohol (Table 1).

**FIGURE 2:**
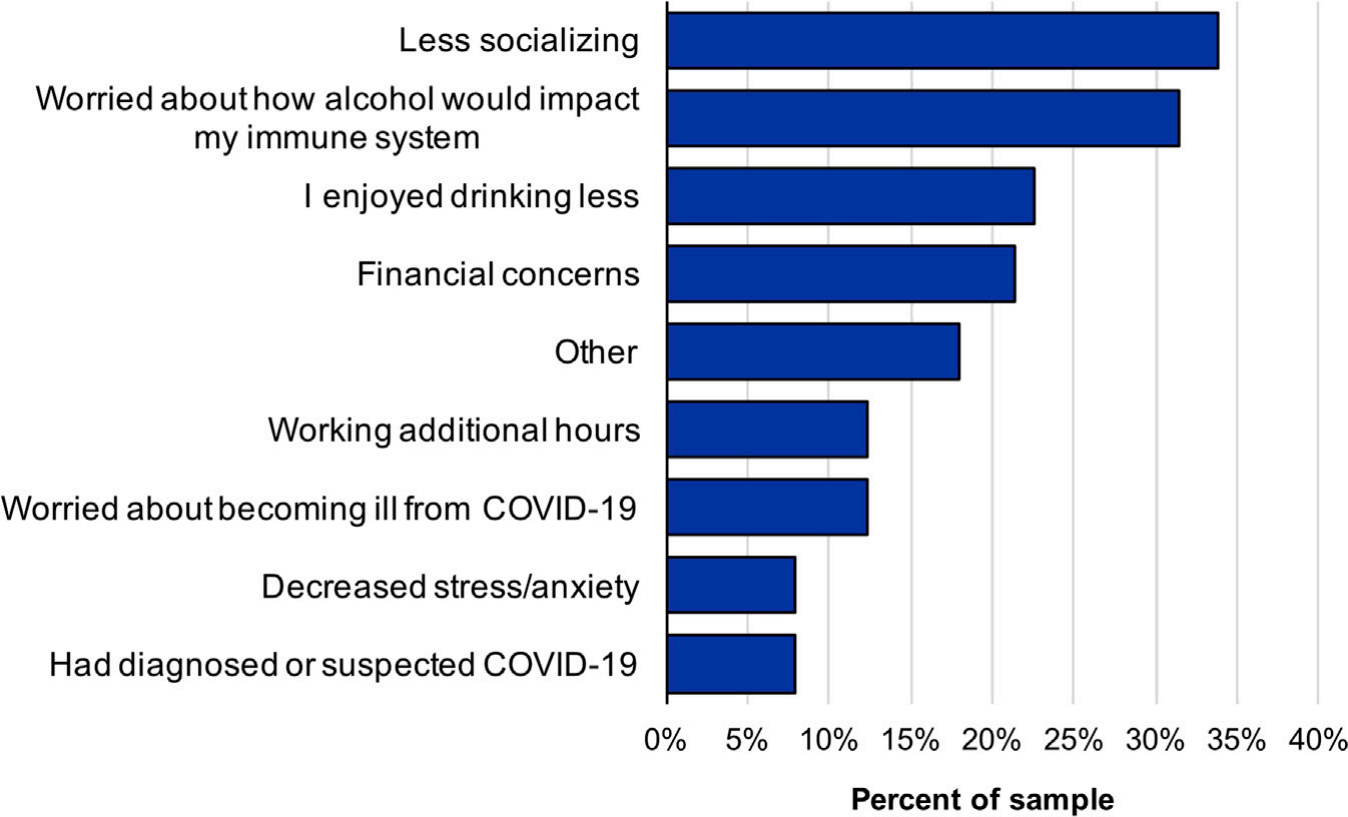
Reasons participants drank less than usual during the COVID-19 pandemic (N = 89).

Fewer participants (*n* = 89) reported drinking less than usual since COVID-19 lockdown began (3.7% very frequently, 6.5% frequently, 22.8% occasionally, 45.1% rarely, and 21.9% never). The most common self-reported reasons for drinking less were less socializing (33.7%) and worrying about how alcohol would impact the immune system (31.5%) (Figure 2). Additionally, among those who reported drinking less, very few selected working additional hours (3%) and financial concerns (6%) as reasons for lowering their alcohol consumption during COVID-19. Statistically significant predictors of drinking less were continued employment and decreased access to alcohol (Table 1).

## Discussion

To our knowledge, this study is the first to assess the impacts of COVID-19 on alcohol consumption in a nationally representative sample of HSA with at-risk drinking. The current study highlighted that female gender, increased self-reported stress, boredom, access to alcohol, and disruption to daily routines may particularly increase susceptibility to elevated alcohol use during a pandemic. Although these factors may heighten the susceptibility to increased alcohol use during social distancing measures (or policies) implemented during a pandemic, these effects are not exclusively the result of a pandemic, these effects are not exclusively the result of a pandemic. These data provide evidence of changes in alcohol use and associated consequences during the COVID-19 pandemic, and suggest that the pandemic has led to greater alcohol use among help-seeking adults attempting to reduce their drinking.

Disasters (i.e., traumatic, natural, or environmental) have often been associated with increases in psychiatric symptoms such as depression and anxiety, and increases in substance use [47]. These conditions were mirrored during COVID-19, particularly during lockdowns throughout the world, where isolation potentially led to alcohol use and misuse in vulnerable individuals [48]. Variations in physiological stress and impaired decision-making increase risk of stress-induced alcohol consumption [48]. The literature on substance use emphasizes the importance of controlling the escalation of alcohol consumption during a crisis and notes two possible scenarios: an increase in consumption due to distress or a decrease in consumption due to the lower accessibility of the substance [26].

Kilian et al. [49] meta-analytical findings suggest that alcohol use declined during COVID-19 in the general population but revealed an increase in alcohol use among those with higher drinking levels prior to COVID-19, confirming the results of the current study. Emerging data during the pandemic has shown associations between anxiety, depression, and psychological distress with drinking among college students [50, 51] and adults globally [13, 52, 53]. Social isolation, boredom, increased availability, and reduced social support may lead to increased drinking [24, 28, 51]. Carlyle et al. [54] findings suggest that certain associates of chance (e.g., anxiety, depression, and resilience) and contextual factors (e.g., loneliness and boredom) contributed to changes in the frequency of use among treatment-seeking individuals during the pandemic. Similarly, among participants who reported drinking more in the present study, some reasons provided by participants were: increased stress/anxiety (65%), boredom, nothing to do at home (60%), spending more time at home (57%), easily became part of my routine’ (47%) and worried about COVID-19 and/or the future (38%). Efforts to overcome boredom, isolation, and psychological distress during a pandemic (e.g., digital interventions, virtual social events, public health education) may be helpful in reducing alcohol consumption.

Past literature suggests two possible mechanisms to explain changes in alcohol consumption in response to economic and employment factors: the first mechanism suggests any increase in alcohol consumption is related to increased situational or psychological distress (i.e., unemployment or loss of salary) and the second mechanism suggests that any decrease in alcohol consumption may be related to decreases in physical and financial availability of alcohol [55]. When asked about how their employment status has been affected by COVID-19, the majority of the participants were either working the same as before (43%) or were able to work from home’ (36%) and only a few were unemployed due to COVID-19 (15%). Further, roughly half (51.2%; 166/324) of the participants reported that their access to alcohol was affected by COVID-19 and 39% reported that their alcohol supply has increased (126/324). Nielsen et al. [56] suggest two possible causal explanations for higher alcohol consumption among those working from home. First, individuals with higher alcohol consumption may choose to work from home to conceal their alcohol use. Second, working from home may facilitate alcohol use that may otherwise not occur [56]. This may be attributed to the relatively high socioeconomic status (SES) of our sample (earned a bachelor’s degree or higher) and a relatively small sample of those who experienced job loss. The findings suggest that any job loss did not occur, or more specifically, to the extent to which it may have influenced a decrease in alcohol consumption.

Emerging literature suggests that women experienced an increase in drinking during the pandemic [24, 57, 58]. During the pandemic, high increases were observed among women when compared to men in terms of frequency (17% versus 11%, respectively), days of heavy drinking (41% versus 7% among men), and alcohol-related consequences [59]. Another study observed increases in heavy-drinking episodes by 41% in women since the COVID-19 lockdown [60]. Our results suggest that Female identifying individuals (*p* = 0.002) were significantly more likely to report drinking more than usual. It is possible that because women are more likely to drink as a result of stress and anxiety than men [61], the consequences of the pandemic put women at greater risk for increased consumption.

To our knowledge, this is the first study to show that the COVID-19 pandemic led to greater alcohol use among HSA attempting to reduce their drinking versus the general population. More research is needed to examine the contexts of drinking during COVID-19 among problem drinkers. Pandemic-like events are expected to increase in the coming decades [62]. The implementation of alcohol prevention strategies in the context of health crises, paying particular attention to individuals working from home and focusing on their reasons and motivations in this specific stress-induced alcohol use context, can help to mitigate the harmful effects of excessive alcohol consumption during challenging times. Identifying and addressing these contextual factors can inform alcohol prevention and control strategies during the current pandemic and may also inform risk and prevention strategies in future pandemics.

## Limitations

This study has several limitations. These findings may not be generalizable to the general population of drinkers due to participants being predominantly female-identifying, White, having bachelor’s degrees or higher (some graduate school or a graduate degree) and being help-seeking for alcohol misuse. Considering that the majority of the participants held a bachelor’s degree or higher may allow increased flexibility in work arrangements, which may have impacted the differences in alcohol consumption [63]. Only self-report questions were used to collect drinking and impacts of drinking data, so the data may be under- or over-reported and subjected to social desirability bias. However, self-report of alcohol use and alcohol use-related behaviors is considered to be relatively valid [64]. Given the cross-sectional design and novelty of the COVID-19 pandemic, the current study limits our ability to make any causal inferences about changes in alcohol consumption prior to and during the pandemic beyond our self-reported question stem “since the pandemic began” anchoring people. Participants were also enrolled in an alcohol intervention study with different treatment groups that received varying levels of intervention. While no differences existed based on treatment conditions, findings may differ in a sample of individuals who were not participating in a treatment study. Lastly, participants’ geographic locations were not accounted for, so the differences in COVID-19 lockdown policies and alcohol sale policies (i.e., to-go alcohol, alcohol delivery services, and liquor stores deemed as essential services) may have influenced alcohol consumption. Despite the highlighted limitations, a novel contribution of the findings distinguishes the impacts of COVID-19 on alcohol consumption among individuals seeking to change their drinking prior to the pandemic.

## Data Availability

The raw data supporting the conclusion of this article will be made available by the authors, without undue reservation.
